# Thanks to Editorial Board of *Arquivos de Neuro-Psiquiatria*

**DOI:** 10.1055/s-0043-1761627

**Published:** 2023-03-14

The Editors of **Arquivos de Neuro-Psiquiatria** thanks to **Associate Editors** (77), **Editorial Board** (50) and **Referees** (312) who altruistically and without payment have expended their time to develop the editorial tasks of this journal since January 1st, 2022 to December 31st, 2022.

Thank you very much!

## Associate Editors

**Table:** 

Adriana Bastos Conforto Alexandra Prufer Queiroz Campos Araújo Ana Carolina Coan Analuiza Camozzato Antonio José da Rocha Antonio Lucio Teixeira Camila Callegari Carlos Henrique Ferreira Camargo Carlos Otto Heise Carolina de Medeiros Rimkus Celi dos Santos Andrade Chien Hsin Fen Clarissa Lin Yasuda Cristiane Nascimento Soares Dalva Lucia Rollemberg Poyares Daniel Ciampi de Andrade Douglas Kazutoshi Sato Edmar Zanoteli Eduardo Genaro Mutarelli Eliasz Engelhardt Elisa de Paula França Resende Ethel Mizrahy Cuperschmid Eva Carolina Rocha Fabio A. Nascimento Fabíola Dach Fernando Morgadinho Santos Coelho Fernando Tensini Francisco de Assis Aquino Gondim Gabriel Rodriguez de Freitas Grace Schenatto Pereira Moraes Michael Hornberger Mônica Sanches Yassuda Octávio Marques Pontes Neto Orlando Povoas Barsottini Paulo José Lorenzoni Paulo Pereira Christo Pedro Andre Kowacs Pedro Sampaio	Iscia Teresinha Lopes Cendes Jamary Oliveira Filho João Brainer Clares de Andrade José Luiz Pedroso Juliana Gurgel-Giannetti Karen Nunez-Wallace Karina Braga Gomes Laura Silveira Moriyama Lea Tenenholz Grinberg Leandro Tavares Lucato Lecio Figueira Pinto Leonardo Cruz de Souza Luciano De Paola Luciene Covolan Luís Otávio Sales Ferreira Caboclo Magda Lahorgue Nunes Marcondes Cavalcante França Jr Marcos Christiano Lange Maria Fernanda Mendes Mariana Spitz Mario Fernando Prieto Peres Marzia Puccioni-Sohler Péricles Maranhão Filho Renato Puppi Munhoz Rosana Cardoso Alves Rosana Hermínia Scola Sarah Teixeira Camargos Sérgio Monteiro de Almeida Sérgio Rosemberg Sheila Cristina Ouriques Martins Sônia Maria Dozzi Brucki Suzana Maria Fleury Malheiros Sylvie Devalle Tarso Adoni Vitor Tumas Vivaldo Moura Neto Wilson Marques Jr. Yara Dadalti Fragoso *(In Memoriam)* Ylmar Correa Neto

## Editorial Advisory Board

**Table:** 

Acary de Souza Bulle Oliveira Alberto J. Espay Alexis Brice Américo Ceiki Sakamoto Andrea Slachevsky Andrew J. Lees Bruce L. Miller Bruce Ovbiagele Carlos Alberto Mantovani Guerreiro Carlos Roberto de Mello Rieder Christina Marra Didier Leys Fernando Cendes Fernando Kok Francisco Eduardo Costa Cardoso Giancarlo Comi Gilmar Fernandes do Prado Henrique Ballalai Ferraz Hugh J. Willison Jaderson Costa da Costa João José Freitas de Carvalho Joaquim Ferreira Joaquim Pereira Brasil Neto José Manuel Ferro Lineu César Werneck	Luiz Henrique Martins Castro Márcia Lorena Fagundes Chaves Marco Aurélio Lana-Peixoto Marcos Raimundo Gomes de Freitas Maria José Sá Maria Lúcia Brito Ferreira Marilisa Mantovani Guerreiro Mônica Levy Andersen Oscar Del Brutto Oscar Gershanik Osvaldo José Moreira do Nascimento Osvaldo Massaiti Takayanagui Pedro Chaná-Cuevas Raimundo Pereira da Silva Néto Regina Maria Papais-Alvarenga Ricardo Allegri Ricardo Nitrini Roger Walz Rubens José Gagliardi Sérgio Teixeira Ferreira Stefan Schwab Ugo Nocentini Umbertina Conti Reed Vladimir Hachinski Walter A. Rocca

## Reviewers

**Table:** 

Abrahão Baptista Acary Souza Bulle Oliveira Adine Adonis Adriana Coitinho Adriana Moro Ailton de Souza Melo Alan Flores Alessandra Zanatta Alessandro Finkelsztejn Alex Meira Alexandra Prufer de Queiroz Campos Araújo Alexandre Fernandes Alfredo Santana Aline Chacon Pereira Allan Brigola Alvisa Palese Amanda Donatti Ana Lucia Rosso Ana Paula Champs Ana Paula Gonçalves Andre Macedo André Silva Angelina Lino Anna Carolyna L. Gianlorenço Antonia Marques-de-Faria Antônio Jaeger Artur Francisco Schumacher-Schuh Auxiliadora Martins Aylin Bican Demir Ayrton Roberto Massaro Breno Barbosa Bruna de Freitas Dias Bruna Dutra Bruno de Oliveira Bruno Pedreira Caio Disserol Camila Pupe Carina Uchida Carla Andrea Caldas Carla Matas Carlos Abanto Carlos Alberto Bordini Carlos Bernardo Tauil Carlos Brandão-Mello Carlos Henrique Camargo Carlos Monteiro Carlos Otto Heise Carlos Roberto Rieder Carlos Rocha Carlos SIlvado Carolina de Medeiros Rimkus Caryne Bertollo Catherine Roch Chin An Lin Cibelle Kayenne Martins Roberto Formiga Clara Camelo Claudete Aparecida Cardoso Claudia Ferreira da Rosa Sobreira Cláudia Maia Memória Claudia Suemoto Cleonísio Rodrigues Conrado Borges Cristiana Borges Pereira Cristiane de Araujo Martins Moreno Cristiane Nascimento Soares Daniel Giansante Abud Daniela M. Zolezzi Daniela Pachito Daniela Pellegrino Danielle Bruno Debora Maia Denise Sisterolli Diniz Diego Fragoso Diogo Santos Eberval Gadelha Figueiredo Eduardo Cavalcanti Eduardo Estephan Eduardo Genaro Mutarelli Eduardo Nicoliche Eduardo Ribas Elcio Juliato Piovesan Elen Beatriz Pinto Eliana Maranhão Elisa de Melo Elisa Resende Elizabeth Comini-Frota Emanuelle Silva Emilio Portaccio Ênio Roberto de Andrade Enrique Gomez-Figueroa Erdem Tüzün Erich Fonoff Ethel Ciampi Eva Rocha Fabiano Abrantes Fabiano Moulin Moraes Fabiano Reis Fabio Ejzenbaum Fabio Godinho Fabrício de Borba Fabrizio Anniballi Farmy Silva Felipe Pacheco Felipe von Glehn Felippe Borlot Feres Chaddad-Neto Fernando Cardoso Fernando Magalhães Fernando Morgadinho Coelho Fernando Stelzer Flávio Rezende Filho Florencia Deschle Francesco Cavallieri Francisco Dias Francisco Gondim Frederico Moura Gabriel Braga Gabriel Cismaru Gabriel Kubota Gabriela Cipolli Gabriele Tonni Gisele Sampaio Silva Givago da Silva Souza Guilherme Baldo Guilherme Sciascia do Olival Gustavo Leite Franklin Gustavo Luvizutto H. Hofstad Hélio A. G. Teive Henrique Ballalai Ferraz Hsin Chien Huseyin Buyukgol Isabella D'Andrea Meira Ivan Aprahamian Jacy Parmera Jamary Oliveira Filho Janini Chen Jefferson Becker Jerusa Smid João Eudes Magalhães Jocemar Ilha Jonas Alex Saute Jorge Bevilacqua José Eduardo Peixoto-Santos José Garbino José Geraldo Speciali José Luiz Pedroso José Marcelino Aragão Fernandes Karen Murphy Karin Zazo Ortiz Katia Lin Katya Naliwaiko Keite Nogueira Kette Dualibi Ramos Valente Laura Silveira-Moriyama Lea Tenenholz Grinberg	Leandro Barbosa Leandro Calia Leandro Iuamoto Leandro Valiengo Lécio Figueira Pinto Léo Coutinho Leonardo Carbonera Leonardo Cruz de Souza Leonardo Sodre Leonardo Welling Leslie Ecker Ferreira Leticia Sampaio Lilian Morillo Liliana Jorge Lisiane Seguti Ferreira Lívia Gitaí Livia Gomes Fregolente Luana Antunes Maranha Gatto Lucas Cabral Lucas Giansante Abud Lucia Nishino Luciene Covolan Luis Henrique Castro Afonso Luiz Claudio Romanelli Luiz Eduardo Betting Luiz Felipe Rocha Vasconcellos Luiz Paulo Queiroz Luiza Perucci Magda Lahorgue Nunes Marcela Freitas Marcelo Aragão Marcelo Masruha Rodrigues Marcelo Matiello Marcelo Ribeiro Caetano Marcelo Riberto Marcia Hallinan Marcio Nattan Marcio Portes Souza Marco Antonio Lima Marco Nihi Marco Pedatella Marcos Christiano Lange Marcos Fábio dos Santos Marcos Gomes de Freitas Marcus Della Coletta Marcus Vinícius Pinto Maria Benevides Maria Ferraz Maria Lucia Vellutini Pimentel Maria Niures Matiolli Maria Sheila Guimarães Rocha Mariana Alves Mariana Spitz Marianna de Moraes Marina Farah Mário Emílio Dourado Mario Habek Mário Luciano de Mélo Silva Júnior Mário Luiz Monteiro Marzia Puccioni-Sohler Matheus Gomes Ferreira Matheus Pedro Mauricio Calomeni Mauro Eduardo Jurno Meire Argentoni Baldocchi Melike Batum Michael Williams Millene Rodrigues Camilo Miriam Menna Barreto Monica Haddad Mônica Parolin Mônica Sanches Yassuda Mustafa Cetiner Nadia Shigaeff Nariana Sousa Nasjla Saba da Silva Nathane Braga Nayanne Bosaipo Norberto Frota Olagide Castro Orlando Graziani Povoas Barsottini Otávio Moreno-Carvalho Pablo Winckler Patricia Coral Patricia Piazza Rafful Paula Sanches Paulo Henrique Ferreira Bertolucci Paulo José Lorenzoni Paulo Pereira Christo Paulo Victor Sgobbi de Souza Pedro André Kowacs Pedro Augusto Sampaio Rocha Filho Pedro Braga-Neto Pedro Castro Pedro Cougo Pedro Kurtz Pedro Naves Pedro Ribeiro Pedro Schestatsky Rafael Carneiro Raphael Spera Raquel Aparecida Casarotto Ravindra K. Garg Raymond Rosales Renata Ducci Renato Puppi Munhoz Ricardo Augusto de Melo Reis Ricardo Silva Pinho Roberta Arb Saba Rodrigo Bazan Rodrigo Mendonca Rogério Aires Ronaldo Abraham Rosalia Otero Rubens Cury Rubens José Gagliardi Saadet Kader Samir Magalhães Sara Freed Sarah Camargos Sebastián Ameriso Sebastian Ortiz Semra Mungan Sergio Cavalheiro Sergio Monteiro de Almeida Sheila Trentin Silvia Takada Silvia Tenembaum Simone Amorim Simone Appenzeller Stefan Schwab Sthella Zanchetta Susan Hillier Tale Aversi-Ferreira Tarsis Vieira Tarso Adoni Tatiane Assone Thiago Cardoso Vale Tiago Aguiar Valeria Scavasine Victor Santos Vinicius Montanaro Vinicius Schoeps Viola Vambol Vitor Paes Vítor Tumas Viviam Sanabria Viviane de Hiroki Flumignan Zétola Wagner Mauad Avelar Wanderley Bernardo Wilfor Aguirre Quispe Wladimir Bocca Vieira de Rezende Pinto Wyllians Borelli Ylmar Correa-Neto Yolanda Maria Garcia Zeferino Demartini Jr

